# Restoring Articulation by Tipping Molars with Gold

**Published:** 1889-07

**Authors:** J. C. Walton


### Restoring Articulation of Tipping Molars with Gold.

J. C. WALTON.

Read before the Michigan State Dental Society, June 6th, 1889.

That any one should ask the attention for a moment or two of such a body on such a subject seems presumptious, yet nowhere in our records as a society, do I find that we have ever given the subject a moment's thought, nor does any literature in my possession mention such an operation, although frequently it is practical and desirable. The object is to bring into occlusion two opposing surfaces of sufficient size to permit your patient in an increased measure to masticate. I say in an increased measure because no repairs by us can promise such perfect results as are accomplished by the proper articulation of perfect teeth. As long as parents continue ignorant or careless of their childrens' dental welfare; as long as first molars continue to decay early, and physicians or dentists unquestioningly extract them, there will be the usual tipping forward of the second molars, thereby destroying that perfect articulation so necessary to perfect mastication.

The observing dentist must repeatedly, in his daily practice, be reminded of how acceptable such an operation will be, and why he should continue to pound gold into cavities of teeth so tipped out of position as to be useless, without attempting to better their articulation, is hard to understand. With this operation, as in all contour work, anchorage is of first importance. Without ample anchorage smooth, strong margins and clean surroundings count for nothing.

In most mouths where the first molars have been out long enough for the consequent changed position of the second molars to offer an excuse for this operation you will find cavities already existing. If none offer a welcome anchorage, then with engine, drill, and burr make liberal retaining grooves, commencing in fissures and cutting with a heroic hand. It is better to bring these teeth into service at such a cost than permit them to remain of no use themselves, and in the way of any appliance that might be serviceable. In the preparation of the crown surface use the corundum wheels freely on cusps, or to prevent thin overlapping edges anywhere. Before beginning the operation notice whether other movements besides tipping have taken place in either upper or lower molars. Aim to have the upper, as a rule, articulate one-quarter or one-third its surface outside the lower to prevent biting the cheek. The articulation of the other teeth must determine whether the articulating surface of the contour is to be a flat surface parallel with the general line of articulation, or an inclined plane. Shape these grinding surfaces so they may be constantly in contact in the act of masticating as the lower jaw moves from side to side. Cut out and fill any depression in a molar that may be in position if necessary to give a good articulation to an opposing contour. Attempt only to secure the millstone effect. Do not attempt the restoration of cusps, for although they are ideal, the material we use is faulty, and no amount of good wishes will add to its strength or wearing qualities.

In filling the excavation use any method or style of gold with which you succeed best. My preference is for heavy foil when strength is of importance, folding back and forth ribbons of No. 60 and making the most of the contour at least of gold and platinum foil shade two. Anneal carefully, condense thoroughly, finish well.

DISCUSSION.

Discussion opened by Dr. Dyer. He had built up tipping molars with gold foil but had found it too soft for the purpose; it wore away rapidly. He preferred restoring the articulation with a cap made of gold coin. He expected to exhibit some practical specimens of his work at the clinic in the morning.

Dr. J. Lathrop had seen some of Dr. Walton's work and considered it very good having withstood mastication for eight years. At Dr. Walton's request he had taken impressions of some work done by him and had brought the models to the meeting for inspection.

Dr. Harroun thought if gold foil was properly manipulated it could be made hard enough to withstand mastication.

Dr. I. Douglas had seen tooth substance worn away faster than the gold filling.

SULPHUR FUMIGATION.—Fumigation by the burning of sulphur is the most common method employed by boards of health in the disinfection of apartments in which contagious disease has existed, and the clothing worn by the patients during their illness. In an address delivered by the distinguished chemist, Dr. E. R. Squibb, before the Kings County Medical Association, he calls attention to the fact that there must always be an abundance of watery vapor in the room to be disinfected ; otherwise the sulphurous acid gas generated by the burning of the sulphur is not an efficient disinfectant. The same is true of chlorine gas when used for disinfecting purposes.—*Science*, April 12, 1889.

